# Mechanisms and Insights for the Development of Heart Failure Associated with Cancer Therapy

**DOI:** 10.3390/children8090829

**Published:** 2021-09-21

**Authors:** Claire Fraley, Sarah A. Milgrom, Lavanya Kondapalli, Matthew R. G. Taylor, Luisa Mestroni, Shelley D. Miyamoto

**Affiliations:** 1 Center for Cancer and Blood Disorders, University of Colorado Anschutz Medical Campus, Aurora, CO 80045, USA; Claire.fraley@childrenscolorado.org; 2 Department of Radiation Oncology, University of Colorado Anschutz Medical Campus, Aurora, CO 80045, USA; sarah.milgrom@cuanschutz.edu; 3 Division of Cardiology, Department of Medicine, University of Colorado Anschutz Medical Campus, Aurora, CO 80045, USA; lavanya.kondapalli@cuanschutz.edu (L.K.); matthew.taylor@cuanschutz.edu (M.R.G.T.); 4 Department of Pediatrics, Division of Cardiology, University of Colorado Anschutz Medical Campus, Aurora, CO 80045, USA

**Keywords:** cardio-oncology, pediatric cancer, cardiotoxicity, anthracyclines, pediatric heart failure

## Abstract

Cardiotoxicity is a well-recognized late effect among childhood cancer survivors. With various pediatric cancers becoming increasingly curable, it is imperative to understand the disease burdens that survivors may face in the future. In order to prevent or mitigate cardiovascular complications, we must first understand the mechanistic underpinnings. This review will examine the underlying mechanisms of cardiotoxicity that arise from traditional antineoplastic chemotherapies, radiation therapy, hematopoietic stem cell transplantation, as well as newer cellular therapies and targeted cancer therapies. We will then propose areas for prevention, primarily drawing from the anthracycline-induced cardiotoxicity literature. Finally, we will explore the role of human induced pluripotent stem cell cardiomyocytes and genetics in advancing the field of cardio-oncology.

## 1. Epidemiology of Cancer Therapy-Related Cardiotoxicity

Heart failure can be an early or late cardiotoxic side effect of cancer treatment in children. Anthracycline cardiotoxicity is characterized as acute if it occurs within the first week of treatment, early-onset progressive cardiotoxicity if it occurs within the first year of treatment completion, and late-onset progressive cardiotoxicity if it occurs more than one year after the completion of treatment [1]. In children, early left ventricular systolic dysfunction may suggest worse outcomes. For example, the Children’s Oncology Group reported that in a study of 1022 pediatric patients with acute myeloid leukemia treated in the AAML0531 clinical trial in which patients received anthracyclines daunorubicin or mitoxantrone, 12% (*n* = 124) developed left ventricular systolic dysfunction over the five-year follow up period. Of these cardiotoxic events, 71% (*n* = 88) were noted during on-protocol therapy and these patients had a 12-fold (95% CI, 4.22–34.8) increased risk of left ventricular systolic dysfunction after treatment completion. Unfortunately, five-year event free survival (HR, 1.57; 95% CI, 1.16 to 2.14, *p* = 0.004) and overall survival (HR, 1.59; 95% CI, 1.15 to 2.19, *p* = 0.05) were significantly lower in patients who developed left ventricular dysfunction. The incidence of cardiotoxicity was higher in noninfants, Black patients, and those with bloodstream infections [2]. A study of Australians who completed treatment for hematologic malignancies found that among the 817 children included, 3.7% (*n* = 30) had an index heart failure admission and 70% of these admissions occurred within one year of the cancer diagnosis, and 83.3% occurred within the first three years of the cancer diagnosis. Of those with heart failure, 53.3% died within the 13-year study period, a nearly four times greater mortality risk than their counterparts without heart failure [3]. With regards to late cardiotoxicity, a study of five-year childhood cancer survivors in the Dutch Childhood Oncology Group—Long-Term Effects After Childhood Cancer cohort showed that the cumulative incidence of heart failure 40 years after childhood cancer was 4.4% (95% CI 3.4 to 5.5%) and that there was a significantly higher cumulative incidence of severe symptomatic heart failure among survivors treated more recently. Young age at time of cancer diagnosis, more recent cancer diagnosis, anthracyclines, mitoxantrone, cyclophosphamide and radiation involving the heart were associated with heart failure risk [4]. Additionally, data from the Childhood Cancer Survivor Study demonstrated that five-year survivors were significantly more likely to report congestive heart failure than siblings (HR 5.9, 95% CI 3.4 to 9.6; *p* < 0.001) [5].

Determining the true prevalence of late cardiomyopathy is complicated by several factors including differences in the definition of cardiomyopathy, screening, study designs, and patient populations. Subclinical disease is generally defined as evidence of abnormalities in cardiac biomarkers, exercise capacity, cardiac function, or arrhythmias in the absence of clinical symptoms. The frequency of subclinical cardiomyopathy in childhood cancer survivors varies widely. In a late term follow-up study of 79 childhood cancer survivors who were 11.2 ± 4.5 past cancer treatment, a third had decreased exercise capacity, 29% had an elevated BNP, and 28% had abnormal strain by echocardiogram [6]. Comparatively, the frequency of symptomatic cardiac dysfunction is as high as 16% [7]. Risk factors for anthracycline-mediated cardiotoxicity include: total cumulative dose, younger age, longer follow up as this results in higher prevalence of cardiac dysfunction, female gender, concomitant mantle radiation, rate of anthracycline administration, and other factors (e.g., concomitant cyclophosphamide, bleomycin, vincristine, amsacrine, or mitoxantrone, trisomy 21, Black race) [1]. A useful tool for predicting the risk of heart failure for a survivor of childhood cancer who has completed cancer treatment within the last five years is the Childhood Cancer Survivor Study (CCSS) Cardiovascular Risk Calculator (as of 1 September 2021; https://ccss.stjude.org/tools-and-documents/calculators-and-other-tools/ccss-cardiovascular-risk-calculator.html).

The incidence of heart failure in children associated with immune checkpoint inhibitors, tyrosine kinase inhibitors, and other targeted cancer therapies has yet to be determined. Retrospective studies of chimeric antigen receptor (CAR)-modified T-cell therapy in children and young adults with hematologic malignancies have reported rates of left ventricular systolic dysfunction of 10–12% [8,9].

## 2. Pathophysiology of Cardiotoxic Cancer Therapies

### 2.1. Anthracyclines

Among antineoplastic agents, anthracyclines are best-known for contributing to cardiotoxicity in childhood cancer survivors. This family of agents includes doxorubicin, daunorubicin, epirubicin, and idarubicin. In a landmark study in adults, Swain et al. reported an estimated cumulative percentage of doxorubicin-related congestive heart failure of 5% of patients who received a cumulative dose of 400 mg/m^2^ of doxorubicin, 26% of patients who received 550 mg/m^2^ of doxorubicin, and 48% of patients who received 700 mg/m^2^ of doxorubicin [10]. Reports from the Childhood Cancer Survivorship study have demonstrated a clear risk above cumulative doses of 250 mg/m^2^ but suggest that there may be no safe dose [5]. While there are many purported hypotheses of anthracyclines’ mechanisms of toxicity, a complete understanding of anthracycline-induced cardiomyopathy does not yet exist. Some of the more common implicated mechanisms will be briefly summarized here.

**Figure 1:**
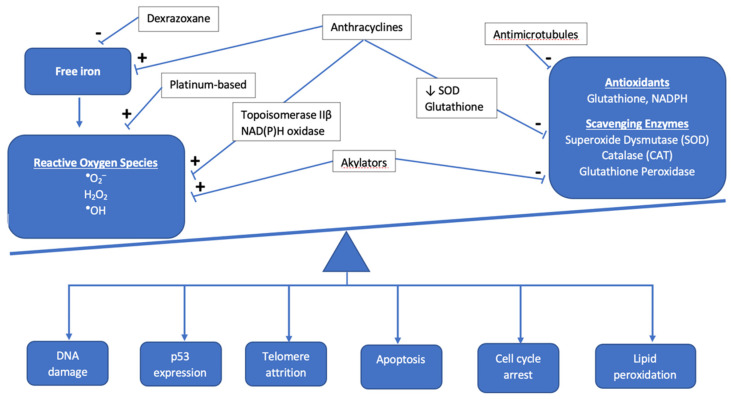
Overview of the interaction between antineoplastic chemotherapy and the role of oxidative stress in development of cardiotoxicity. The cumulative effect of chemotherapeutic agents results in the accumulation of reactive oxygen species and other mediators of oxidative stress, such as free iron, as antioxidants and scavenging enzymes are prevented from maintaining proper balance as they normally do under healthy physiologic conditions.

Anthracyclines increase cellular oxidative stress, which is associated with an imbalance between the creation of reactive oxygen species (ROS) and scavenging enzymes or antioxidants that normally keep ROS in check (Figure 1). Elevated ROS leads to oxidative stress as electrons are scavenged to reach a more stable state, leading to DNA damage, lipid membrane peroxidation, cell cycle arrest, telomere attrition, and apoptosis [11]. The most commonly used anthracycline in pediatric oncology is doxorubicin, which exerts its anti-neoplastic effect via topoisomerase IIα binding to DNA, inducing cell death. While tumor cells are rich in topoisomerase IIα, cardiomyocytes exhibit the topoisomerase IIβ isoform, which is thought to regulate genes involved in mitochondrial biogenesis and function [12]. Under doxorubicin’s influence, oxidative phosphorylation within the mitochondria is impacted and leads to an imbalance in favor of increased ROS, ultimately leading to cardiotoxicity [13]. Mitochondrial dysfunction also results from anthracyclines forming complexes with the inner mitochondrial membrane phospholipid cardiolipin, thereby disrupting electron transport chain activity and resulting in a shift to increased reliance on glucose metabolism in cardiomyocytes, which is a final common pathway in heart failure [14,15].

In addition to oxidative stress, there are other mechanisms of cellular apoptosis and cell injury mediated by anthracyclines. Anthracyclines cause direct DNA damage through the cleavage of DNA strands, but they also inhibit DNA biosynthesis and the enzymes involved in DNA repair [16]. Anthracyclines increase the likelihood that calcium channels in the sarcoplasmic reticulum are in the open state, resulting in an increase in calcium release [17,18]. Sustained calcium leak leads to further ROS production and ultimately culminates in the activation of the caspase cascade and cellular apoptosis. Anthracyclines result in impaired iron sequestration and, therefore, an increase in free iron accumulation in cardiomyocytes and mitochondria, further contributing to free radical generation, cell damage, and mitochondrial dysfunction [19]. Anthracyclines also impair nitric oxide and endothelin-1 production, contributing to endothelial cell dysfunction and diminished cardiomyocyte survival [20].

Genome wide association studies (GWAS) have contributed to the identification of possible genetic modifiers of risk for the development of anthracycline-induced cardiomyopathy; they are summarized in Table 1. In a recent review, Magdy and colleagues summarized the single nucleotide polymorphisms (SNPs) that increase the risk of cardiotoxicity [21]. For example, at the enzymatic level, the S427L variant of the *RARG* gene increases topoisomerase IIβ expression and is associated with worse cardiac outcomes among pediatric cancer survivors [22]. Variants in NAD(P)H oxidase subunits have been proposed to result in both acute and chronic forms of cardiotoxicity via ROS formation; [23,24,25] Cascales et al. examined cardiac histology among recently deceased patients treated with anthracyclines and discovered a five-fold increased odds (95% CI: 1.59–16.43) of cardiac interstitial fibrosis in the presence of the rs1883112 SNP in the p40phox subunit of NAD(P)H oxidase [26]. However, the rs4673 SNP in the p22phos subunit of NAD(P)H showed mixed results. While Cascales and colleagues noted a protective effect for those with the rs4673 SNP against myocardial fibrosis in the decedent samples (OR 0.11, 95% CI: 0.2–0.63), Wojnowski et al. found an association of this SNP with acute cardiotoxicity [23,26]. Moving from the enzymatic to the sarcomeric level, variants in the titin truncating gene (*TTN*) have been found to occur at higher rates in patients with cardiotoxicity than in the general population [27]. Garcia-Pavia subsequently confirmed the development of cardiomyopathy in *TTN* variant mice treated with anthracyclines [27].

Cardiotoxicity is also thought to arise from alterations in anthracycline transport and metabolism. In Magdy et al.’s review of the literature, they approximated that 45% of SNPs implicated in cardiotoxicity are associated with drug transport, which largely take the form of regulating the concentration of doxorubicin and its metabolites intracellularly [21]. A haplotype that is particularly salient in the pediatric literature is the *UGT1A6* gene involved in drug glucuronidation. The UGT1A6*A haplotype is associated with a predictable decrease in enzyme activity, which is thought to lead to slower drug and metabolite clearance [28,29]. Although less well studied, alterations in sarcomeric genes may play a role in anthracycline-induced cardiotoxicity. Investigations of patients with anthracycline induced cardiotoxicity in the setting of a family history of dilated cardiomyopathy have found variants in the *MYH7* gene to be implicated [30]. This same gene was shown to be downregulated in human induced pluripotent stem cell-derived cardiomyocytes (hiPSC-CM) treated with doxorubicin, identifying this gene as a potential target for future study [31]. While these studies suggest a potential role for genetics and SNPs in identifying those most vulnerable to cardiotoxicity, this line of investigation requires further validation, particularly in the pediatric population.

### 2.2. Non-Anthracycline Agents

Many non-anthracycline chemotherapy agents are thought to exert their cardiotoxic effects via oxidative stress as well, though at much lower rates than their anthracycline counterparts. For example, a recent review, Zhang and colleagues summarize studies that suggest cyclophosphamide and its metabolites have the potential to both increase ROS production and interfere with the antioxidant system [32]. Cisplatin has been found to cause an accumulation of ROS, while anti-microtubule agents like vinblastine have been shown to decrease the activity of ROS scavenging enzymes [32,33].

Endothelial damage is thought to be a mechanism of cardiotoxicity mediated by non-anthracyclines as well. There is evidence that alkylators like cyclophosphamide directly damage endothelial cells, allowing for the extravasation of toxic metabolites and direct damage to myocytes [34]. While cardiotoxicity from 5-fluorouracil (5FU) is rare, it can occur and take the form of acute coronary syndrome, which may be mediated by nitric oxide synthase (NOS) causing coronary artery spasm, endothelium-dependent vasoconstriction, and ultimately, ischemia [35,36]. Finally, thromboembolism formation may be promoted by agents like cisplatin and cyclophosphamide, similarly predisposing to ischemia [34].

### 2.3. Radiation-Induced Cardiotoxicity

#### 2.3.1. Mechanisms for Toxicity

Radiation therapy (RT) has improved oncologic outcomes for many pediatric cancers; however, RT to the chest is associated with a risk of cardiotoxicity. Radiation induces DNA damage, oxidative stress, endothelial cell senescence, and pro-inflammatory pathways. These changes may result in intimal thickening, fibrin deposition, lipid accumulation, and thrombosis [37]. In the acute time period, pericarditis and myocarditis may be observed; however, their incidence is low with modern RT. Late effects, such as coronary artery disease (CAD), valvular disease, restrictive cardiomyopathy, and arrhythmias may become apparent years or decades after RT [37,38]. Importantly, the age at onset of late effects is variable and depends in part on the patient’s age when cancer therapy commenced. While the median age of onset of cardiac disease in those exposed to radiation and other cardiotoxic therapies is generally in young adulthood, myocardial infarctions, congestive heart failure, pericardial disease, and valvular abnormalities can occur in the childhood years [5].

Other treatment and patient-specific factors increase the risk of radiation-associated cardiotoxicity. For example, the incidence of cardiotoxicity is higher in patients who received both anthracycline-based chemotherapy and RT compared to RT alone [39,40]. In addition, conventional risk factors, such as dyslipidemia, hypertension, and smoking, are independently associated with late cardiotoxicity [41,42,43,44]. Therefore, guidelines recommend regular screening for modifiable cardiac risk factors and the initiation of appropriate interventions in survivors who received RT to the chest [45].

#### 2.3.2. Dose-Toxicity Relationship

Radiation-specific factors that influence the risk of cardiotoxicity include the total radiation dose to the heart, the volume of the heart exposed, and the dose per fraction [40,41,42,43,44,46,47] (Table 2). Recent data from the Childhood Cancer Survivor Study demonstrated a significant association between mean heart dose (MHD) and multiple adverse cardiac outcomes (heart failure, CAD, valvular disease, arrhythmia) in multivariable models that accounted for sex, treatment decade, anthracycline dose, and relevant comorbidities [41]. Importantly, this same study demonstrated that both the MHD and the risk of CAD in long-term survivors declined significantly over the study period. The decreased incidence of CAD with treatment decade was attenuated by adjustment for cardiac radiation exposure, suggesting that more modern RT is associated with a lower risk of CAD than historic RT [41].

### 2.4. Targeted Cancer Therapies

Targeted chemotherapy is more widely used in pediatric cancer, as our understanding of specific mutations in cancer development and the immune regulation to keep tumors in check is advanced. These therapies include immune checkpoint inhibitors, tyrosine kinase inhibitors (TKIs), and proteasome inhibitors. While there is great optimism for their role in revolutionizing cancer therapy, they are not without side effects, including cardiotoxicity.

**Figure 2:**
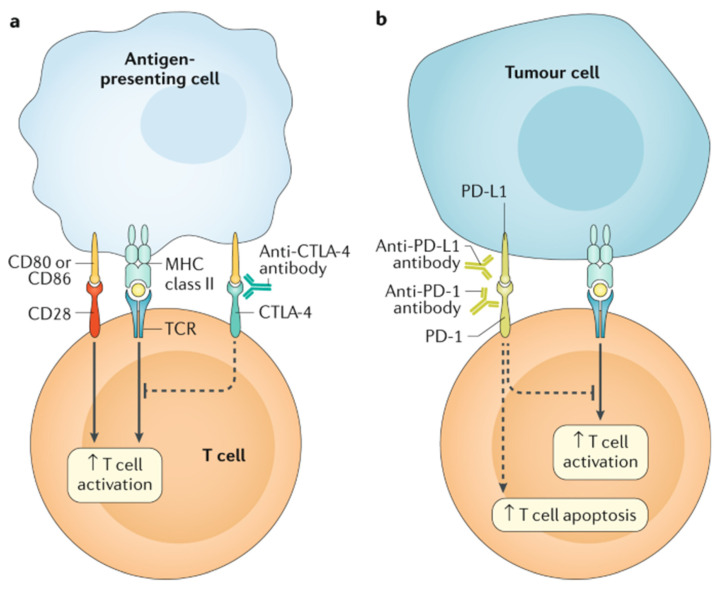
The main immunotherapy approaches that are approved for clinical use in cancer are the immune checkpoint inhibitors. These therapies are monoclonal antibodies that target the CTLA-4 and PD-1 receptors and the PD-1 ligand PD-L1, which are involved in the regulation of T cell activation. (**a**) T cell activation requires two signals: first, antigen recognition by the T cell receptor (TCR) following antigen presentation by major histocompatibility complex (MHC) class II molecules on the surface of antigen-presenting cells; and, second, signal modulation by CD80 or CD86 binding to the CD28 receptor. CTLA-4 is located on the T cell surface and competes with the CD28 receptor to bind CD80 or CD86, thereby blocking T cell activation. CTLA-4 inhibitors block CTLA-4–CD80 or CTLA-4–CD86 binding to facilitate T cell activation (dashed line). (**b**) PD-1 is a surface receptor that is expressed by T cells and promotes apoptosis of antigen-specific T cells and reduces apoptosis of regulatory T cells through its interaction with its ligand, PD-L1, which is expressed by tumour cells and myeloid cells. This interaction is useful in preventing autoimmunity in physiological conditions, but cancer cells exploit this process to escape from immune system activity upregulating PD-L1 expression. PD-1 and PD-L1 inhibitors block the PD-1–PD-L1 interaction, facilitating T cell activation and survival (dashed lines). (Figure with permission from Ramos-Casals M, Brahmer JR, et al. Immune-related adverse events of checkpoint inhibitors. Nat Rev Dis Primers. 2020 May 7;6(1):38).

Immune checkpoint inhibitors work by blocking tumors’ attempts at evading the immune system, primarily by re-engaging T cell detection of tumors (this concept is further demonstrated in Figure 2). Tumors learn to express cytotoxic T-lymphocyte antigen 4 (CTLA-4), programmed cell death 1 (PD-1), and the ligand for PD-1 (PD-L1), all of which downregulate the immune response. Monoclonal antibodies against these receptors antagonize these downregulatory functions (effectively turning off the off switch) and allow the immune system to join in the fight against malignancy [50]. Examples include ipilimumab against CTLA-4 and nivolumab and pembrolizumab against PD-1.

Animal models suggest that the mechanistic underpinnings of cardiotoxicity associated with these agents may be a consequence of their activation of the immune system. For example, when CTLA-4 or PD1 is deleted in mouse models, autoimmune myocarditis and even dilated cardiomyopathy occur [51,52,53]. Furthermore, in mouse models examining T-cell mediated myocarditis, PD-L1 has been found to be upregulated, suggesting a protective effect [54].

Among TKIs, the most commonly used in pediatrics are mitogen-activated protein kinase kinase (MEK) inhibitors (e.g., trametinib, cobimetinib) as well as vascular endothelial growth factor (VEGF) inhibitors (e.g., sorafenib, bevacizumab). It is well established that VEGF inhibitors carry an increased risk for the development of hypertension, thereby increasing risk of heart failure if blood pressures are not adequately controlled [55]. In addition, there is some evidence that VEGF inhibitors lead to cardiotoxicity by decreasing capillary density and targeting hypoxia inducible factor-1-alpha (HIF-1α), which leads to myocardial hypoxia and cardiac dysfunction [56]. This is further supported by the development of cardiomyopathy in mice overexpressing HIF-1α [57].

In the adult literature, trastuzumab, an anti-human epidermal growth factor receptor 2 (HER2) monoclonal antibody used for the treatment of HER2 positive breast cancer, is widely known to contribute to cardiotoxicity [12,21,58,59]. Trastuzumab was briefly used for HER2 positive osteosarcoma in pediatric populations and was found to be well tolerated but did not significantly improve overall survival [60]. Since then, it has fallen out of favor in pediatric populations given its lack of efficacy and potential for cardiotoxicity.

### 2.5. Cellular Therapy and Hematopoietic Stem Cell Transplantation

Many childhood diseases, malignant and otherwise, are seeing improved survival rates as a result of allogenic or autologous bone marrow transplantation. The cardiotoxicity experienced in this population of children varies according to the risk factors they carry prior to transplant as well as their experiences post-transplant. Among patients transplanted for malignancies, many children are placed at a higher risk for developing cardiotoxicity due to pre-treatment with high doses of anthracyclines to induce a remission prior to their consolidative transplant [61]. Patients transplanted for non-malignant conditions, such as sickle cell disease or thalassemia, often receive repeated red blood cell transfusions and are thus at higher risk for iron overload. Iron can deposit in the myocardium, increasing this population’s risk for eventual heart failure [61]. Finally, malignant and non-malignant patients alike may receive total body irradiation as a part of their preparative regimen for bone marrow transplantation, increasing their risk for cardiotoxicity for the reasons mentioned previously [61].

A number of factors that arise post-bone marrow transplantation increase the risk for cardiotoxicity as well. Patients are most often treated with calcineurin inhibitors (e.g., cyclosporin) as prophylaxis for graft versus host disease (GVHD). Calcineurin inhibitors are associated with systemic hypertension, and if blood pressures are not adequately managed, can increase the risk for development of heart failure [61]. If GVHD does occur, it is often treated with corticosteroids, sometimes for long periods of time. The side effect profile of corticosteroids includes hypertension, hyperglycemia, and weight gain, which are all risk factors for heart disease [61]. Finally, there is a higher incidence of metabolic syndrome in the post-transplant population, which compounds the risk factors that are already present [61]. While development of heart failure as a consequence of these modifiable risk factors can take years or even decades to develop, the appropriate identification and treatment of these issues in the childhood years are critical to mitigate future cardiovascular risk and improve longer term outcomes.

Cellular therapy is an evolving field for treatment of childhood cancers. Most promising thus far is the development of chimeric antigen receptor (CAR) T cells. To engineer a CAR-T cell, a patient’s T cells are collected via leukapheresis, injected with a genetically engineered receptor to target antigens on the surface of tumor cells, expanded, and infused back into the patient. This was first used in pediatric pre-B acute lymphoblastic leukemia (ALL), where CD-19 on the surface of B cells was targeted by CAR-T cells [62]. Patients treated with CAR-T therapy are at risk for cardiotoxicity both because of treatment they may have seen in the past (e.g., anthracyclines) in addition to acute risks associated with the CAR-T cells, most notably cytokine release syndrome (CRS).

CRS is a phenomenon in which the infused CAR-T cells stimulate both the innate and adaptive immune system, releasing supraphysiologic levels of cytokines leading to systemic inflammation [63]. CRS ranges from mild to severe, with signs and symptoms ranging from fever and tachycardia to hypoxia, hypotension, and, in the worst case, organ failure and death. IL-6 is an important mediator of CRS and is released by antigen presenting cells (APCs) and activated endothelial cells [50]. The dysfunction of endothelial cells then contributes to vascular leak and disruption of the blood brain barrier, leading to CAR-T cell induced neurotoxicity (i.e., immune effector cell associated neurotoxicity/ICANS). IL-6 is thought to be secreted at the same time CAR-T cells are recognizing antigenic targets on tumor cells, not as a direct result of CAR-T cells engaging APCs or endothelial cells [50]. The role of IL-6 in CRS is further supported by the rapid improvement seen with the initiation of tociluzimab, a monoclonal antibody against the IL-6 receptor [64].

Acute cardiotoxicity in the setting of CAR-T cell therapy occurs almost exclusively in the setting of CRS and most closely resembles cardiomyopathy associated with sepsis [50]. IL-6 is largely to blame for cardiotoxicity in this context, which is consistent with previous work showing this cytokine to be implicated in myocardial depression in both inflammatory and infectious states [65]. Supraphysiologic levels of this and other pro-inflammatory cytokines can lead to tachycardia, hypotension, troponin elevation, reduced left ventricular ejection fraction, pulmonary edema, and cardiogenic shock [50].

Finally, metabolic derangements can play a role in CAR-T cell associated cardiotoxicity. When CAR-T cells engage target antigens on the surface of tumor cells and induce cell lysis, intracellular electrolytes such as potassium, phosphate, calcium, and uric acid spill into the extracellular space in a phenomenon known as tumor lysis syndrome. These metabolic derangements may lead to arrythmia and renal failure if left unchecked [50].

## 3. Prevention

### 3.1. Drug Delivery

Preventing cardiotoxicity among childhood cancer survivors includes optimizing the delivery of antineoplastic chemotherapy and irradiation in addition to minimizing oxidative stress. For example, a recent clinical practice guideline recommended anthracyclines be administered over at least an hour; this was associated with decreased cardiotoxicity with preserved tumor response and overall survival when compared to anthracycline administered as a bolus [66].

Altering the delivery of anthracyclines has also taken the form of liposomal encapsulation. The motivation for this delivery method came from the idea that liposomes remain confined to the intravascular space in areas with tight capillary junctions (e.g., the myocardium), but are able to extravasate into tissues without tight endothelial junctions, such as tumors, thereby preferentially directing liposomes towards the tumor and away from healthy tissue that are vulnerable to insult [67] The three liposomal anthracyclines that have received the most attention are liposomal doxorubicin (DaunoXome^®^), nonpegylated liposomal doxorubicin (Myocet^®^), and pegylated liposomal doxorubicin (Doxil^®^), though attention is now being turned to CPX-351, a liposomal preparation of cytarabine and daunorubicin. CPX-351 has been incorporated into the ongoing AAML1831 trial, a Children’s Oncology Group study for the treatment of Acute Myeloid Leukemia. Given CPX-351’s success for children with relapsed AML [68], the study authors have incorporated it into this open trial of up-front therapy to evaluate acute and late cardiac effects compared to non-liposomal anthracyclines [69]. 

### 3.2. Dexrazoxane

Dexrazoxane has been incorporated into anthracycline-containing chemotherapy regimens to minimize oxidative stress. Dexrazoxane is an iron chelator and exerts its effect by scavenging free iron that accumulates as a result of anthracyclines, thereby reducing ROS production. Long term follow-up from the Pediatric Oncology Group 9404 protocol found that concomitant dexrazoxane administration in patients with T cell acute lymphoblastic leukemia and other lymphoblastic non-Hodgkin’s lymphomas improved fractional shortening measured on echocardiogram while maintaining overall survival rates [70]. Furthermore, a meta-analysis and systematic review conducted by Shaikh and colleagues examined the effect of dexrazoxane on development of cardiotoxicity in children [71]. The authors demonstrated that, when examined in the context of randomized clinical trials (RCTs), dexrazoxane did not affect cardiotoxicity given the low event rates. However, when examined in the context of nonrandomized studies, dexrazoxane reduced the risk of clinical or subclinical cardiotoxicity by more than half (RR = 0.29–0.43) [71]. Historically, there were concerns that dexrazoxane may increase risks of infection, myelosuppression, and secondary primary malignancies. However, subsequent studies have demonstrated that the use of dexrazoxane is safe, and does not increase the risk of secondary malignancies, does not impact chemotherapy efficacy, and does not compromise event free or overall survival [72,73,74]. Dexrazoxane is the only FDA-approved drug for preventing anthracycline induced cardiomyopathy and it is reasonable to consider the use of dexrazoxane as a cardioprotective strategy for patients being treated with anthracycline containing regimens such as osteosarcoma, lymphomas, Wilms tumor, and childhood leukemias [72,75,76,77].

### 3.3. Evolution in RT Treatment Planning to Reduce Cardiac Dose

Numerous advances have contributed to reducing cardiac exposure with modern RT. First, with current diagnostic imaging and systemic therapy, irradiated volumes are often smaller than they were with historic approaches; for example, in Hodgkin lymphoma (HL), total lymphoid irradiation and mantle fields have been replaced by targeted, individualized “involved site RT” [78]. Furthermore, technological developments in radiation oncology improve cardiac sparing. Advanced radiation techniques, such as intensity modulated RT and proton beam therapy, deliver highly conformal radiation dose, minimizing the exposure of adjacent normal tissues. Image guidance at the time of RT enables the use of smaller margins to account for uncertainty in patient positioning. Modern thoracic RT may be delivered with a deep inspiration breath hold technique that reduces dose to the heart and lungs [78,79]. Advancements such as these have resulted in a marked reduction in cardiac exposure. For example, in HL, the MHD was typically ≥35 Gy with historic techniques [41]; in contrast, it is typically in the single digits with a modern RT approach [80].

## 4. Future Directions

The identification of novel model systems are critical in the quest to advance our understanding of mechanisms of cancer therapy-induced cardiotoxicity. The use of patient-specific hiPSC-CMs is being explored as an avenue to risk stratify patients with respect to chemotherapy-induced cardiotoxicity. This “clinical trial in a dish” can be leveraged to study the implication of SNPs and investigate the underlying mechanisms resulting in differing responses to drugs [81]. Susceptibility to cardiotoxicity in populations of cancer patients has been revealed through study of hiPSC-CMs isolated from patients with clinical evidence of doxorubicin-induced cardiotoxicity compared to those without cardiotoxicity [31]. hiPSC-CMs of those patients with cardiotoxicity demonstrated decreased cell viability and impaired mitochondrial and metabolic function, as well as altered calcium handling and increased ROS production. Another study demonstrated that the treatment of hiPSC-CMs from healthy subjects with trastuzumab resulted in impaired contractility and calcium handling [82]. Importantly, hiPSC-CMs from cancer patients who experienced trastuzumab associated cardiac dysfunction were more severely affected than hiPSC-CMs from patients who did not have cardiac dysfunction. These studies and others offer an exciting new landscape for studying the mechanisms of cardiotoxicity in vitro while still closely resembling in vivo environments. While pharmacogenetic testing to inform chemotherapy dosing is not yet a mature practice, it may be an important application in the field of oncology in the future.

## 5. Conclusions

There is an evolving body of literature exploring the mechanisms involved in cardiotoxicity related to cancer therapies. It is critically important to advance our understanding of these mechanisms so that cancer therapies can be better tailored to effectively treat cancer, while simultaneously limiting potentially life-limiting and life-threatening complications. In addition, there are promising data using hiPSC-CMs for risk stratification of individual patients, as well as patient populations with respect to cardiotoxic risk. The consideration of cardiotoxicity in pediatric cancer patients is arguably of even greater importance given the anticipated post-cancer lifespan of these children. Therefore, dedicated studies of cardiotoxicity mechanisms and opportunities to mitigate this risk in children are needed.

## Figures and Tables

**Table 1:** Overview of genetic modifiers of cardiotoxicity risk.

Genetic Modifiers of Cardiotoxicity Risk	
**Deleterious Effect**	
**Gene/SNP**	**Mechanism of toxicity**
*RARG* (S427L variant)	Increase topoisomerase IIβ expression
rs1883112 SNP, p40phox subunit NAD(P)H oxidase	Interstitial fibrosis
UGT1A6*A	Decreased drug glucuronidation and clearance
*MYH7*	Variants documented in familial dilated cardiomyopathy Downregulated in hiPSC-CM treated with doxorubicin
Titin truncating variants (*TTN*)	Encode A and I bands in sarcomeres; associated with depressed LV function
** Indeterminant Effect**	
rs4673 SNP, p22phos subunit NAD(P)H	Protective against myocardial fibrosis Acute cardiotoxicity

**Table 2:** Studies exploring the association of radiation dose with risk of grade ≥ 3 cardiac toxicity in adult survivors of childhood cancers. The reference group comprised adult survivors or childhood cancers without the cardiotoxic exposure. Higher radiation dose to the heart was associated with an increased risk of grade ≥ 3 cardiotoxicity. The risk was higher in patients who received anthracycline chemotherapy in addition to radiation therapy.

First Author	n	Treatment Period	Age at Treatment, Years	Length of Follow-Up, Years (Median)	Risk of Grade ≥ 3 Cardiotoxicity (95% CI)
Haddy [48]	3162	1942–1985	<17	26	**No anthracycline:** 1–5 Gy: RR 1.9 (0.4–8.8) 5–15 Gy: RR 4.6 (1.1–19.4) 15–30 Gy: RR 19.5 (5.6–67.8) >30 Gy: RR 75.2 (21.6–1261.2) **Anthracycline:** 1–5 Gy: RR 40.9 (10.9–153.0) 5–15 Gy: RR 37.5 (8.7–161.7) 15–30 Gy: RR 59.5 (16.9–209.6) >30 Gy: RR 110.7 (28.4–432.4)
van der Pal [49]	1362	1966–1996	<18	22	**Radiation only:** HR 13.0 (2.8–61) **Anthracycline & radiation:** HR 49.5 (10.7–230) HR 1.8 (1.4–2.2) per 10 Gy to heart
Mulrooney [41]	23,462	1970–1999	<20 (median 6)	28	**HF:** 1–15 Gy: 0.74 (0.54–1.03) 15.1–34.99 Gy: 1.56 (1.05–2.33) ≥35 Gy: 3.95 (2.87–5.43) **CAD:** 1–15 Gy: 1.31 (0.88–1.96) 15.1–34.99 Gy: 2.26 (1.32–3.84) ≥35 Gy: 5.86 (3.69–9.28) **Valvular disease:** 1–15 Gy: 1.12 (0.33–3.79) 15.1–34.99 Gy: 2.03 (0.64–6.44) ≥35 Gy: 13.97 (6.01–32.48) **Pericardial disease:** 1–15 Gy: 0.64 (0.19–2.20) 15.1–34.99 Gy: 0.88 (0.21–3.64) ≥35 Gy: 2.77 (0.59–12.88) **Arrhythmia:** 1–15 Gy: 0.97 (0.54–1.73) 15.1–34.99 Gy: 0.89 (0.37–2.18) ≥35 Gy: 2.74 (1.10–6.81)

CAD: coronary artery disease; HF: heart failure; HR: hazard ratio; RR: relative risk.
